# USE IT TOO MUCH AND LOSE EVERYTHING? THE EFFECTS OF HOURS OF WORK ON HEALTH

**DOI:** 10.1093/geroni/igz038.3418

**Published:** 2019-11-08

**Authors:** Colin McKenzie, Kei Sakata, Shinya Kajitani

**Affiliations:** 1 Keio University, Tokyo, Japan; 2 Australian Institute of Family Studies, Southbank, Victoria, Australia; 3 Kyoto Sangyo University, Kyoto-shi, Kyoto-fu, Japan

## Abstract

We examine the causal impact of working hours on various health outcomes of Australian men aged 40 and over. To capture the potential non-linear dependence of health status on working hours, the models for health outcomes include working hours and its square. We deal with the potential endogeneity of working hours by using the instrumental variable estimation technique using instruments based on the age for pension eligibility. A non-linear causal effect of working hours on health is confirmed. For males working relatively moderate hours (up to 20–24 hours for a week), an increase in working hours has a positive impact on health, but thereafter an increase in working hours has a negative impact on health. The results also indicate that there is a non-linear dependence of working hours on the pension eligibility age, and also a non-linear dependence of health outcomes on the pension elgibility when this last relationship is treated as a "reduced form" relationship.

